# The complete chloroplast genome and phylogenetic analysis of *Astragalus scaberrimus* Bunge 1833

**DOI:** 10.1080/23802359.2021.1997108

**Published:** 2021-11-10

**Authors:** Yupeng Guo, Buqing Yao, Mengran Yuan, Junqiao Li

**Affiliations:** a Qinghai Provincial Key Laboratory of High value Utilization of Characteristic Economic Plants, The College of Ecological Environment and Resources, Qinghai Minzu University, Xining, Qinghai, P. R. China; b Key Laboratory of Cold Regions Restoration Ecology, Qinghai Province, and Key Laboratory of Adaptation and Evolution of Plateau Biota, Northwest Institute of Plateau Biology, Chinese Academy of Sciences, Xining, Qinghai, P. R. China

**Keywords:** *Astragalus scaberrimus* Bunge, chloroplast genome, phylogenetic analysis

## Abstract

*Astragalus scaberrimus* Bunge 1833 is a widespread perennial herb in northern China. The plant has white flowers and white hairs on the leaves and stems. To determine the chloroplast genome, total DNA was extracted from a sample and sequenced on the Illumina HiSeq4000 platform. After sequencing, the reads of chloroplast DNA were assembled and annotated via NOVOPlasty and PGA respectively. The chloroplast genome of this plant has a circular form with a length of 123,492 bp, a 34% GC content and IR loss. After annotation, a total of 113 genes were predicted for this cp genome, comprising 79 encoded proteins, 4 rRNAs and 30 tRNAs. The evolutionary history indicates that *A. scaberrimus* was grouped within *Astragalus* and formed a clade with *Astragalus laxmannii* with a 100% BS support value. The complete chloroplast genome can serve as a reference for future studies on molecular biology, evolution, population genetics, taxonomy and resource protection.

*Astragalus scaberrimus* Bunge 1833, belonging to Fabaceae, is a perennial herb, 8–15 cm tall, acaulescent to more rarely shortly caulescent, leaflets in 3–6 pairs, narrowly elliptic to elliptic, little white racemes loosely 3–5 flowered, and the surface of leaves and stems covered with appressed white hairs. The plant is widespread in northern China, such as in Gansu, Hebei, Heilongjiang, Henan, Jilin, Liaoning, Nei Mongol, Ningxia, and Qinghai (Delectis Florae Reipublicae Popularis Sinicae Agendae Academiae Sinicae Edita 1993). Some species of *Astragalus* have bioactive constituents, and crude extracts of *Astragalus* have been reported to have anti-inflammatory, immunostimulant, anticancer, antioxidative, cardioprotective, and antidiabetic functions (Keith et al. 2003; Li et al. 2014). The dried roots of some species of *Astragalus* have been successfully used in traditional Chinese medicines to cure empyrosis, nephritis, diabetes mellitus, hypertension, cirrhosis, leukemia, uterine cancer, etc. (Li et al. 2014; Zhang et al. 2019). Among the many studies that have been conducted on the phytochemistry and pharmacology of *Astragalus* plants, only a few studies have been conducted on *A. scaberrimus*, including a number and karyotype analysis (Yang and Sheng 2002), new taxa identification (Jiang and Yin 1992) and a study on phytoremediation of saline-alkali wastelands (Zhang et al. 2013). So far, this plant is used mainly as a forage grass and is considered a soil-and-water-conservation plant. Here, we report the complete chloroplast (cp) genome and analyze its phylogenetic relationship with other related species.

A few samples were collected from the Qilian Mountains (36°34′32″N, 101°48′43″E) in Qinghai Province. Total genomic DNA was extracted from the fresh leaves of a sample with a Rapid Plant Genomic DNA Isolation Kit (Sangon Biotech (Shanghai) Co., Ltd.). A specimen under voucher number HCEERQNU-20200503011 and total DNA under number Astragalus-sca-DNA01 were deposited at the College of Ecological Environment and Resources, Qinghai Nationalities University (https://shxy.qhmu.edu.cn/, Junqiao Li, email: ljqlily2002@126.com). Paired-end libraries with an average length of 500 bp were constructed and sequenced on the Illumina HiSeq4000 platform (Sangon Biotech (Shanghai) Co., Ltd.). The complete cp genome was assembled via NOVOPlasty 3.7.2 (Dierckxsens et al. 2017) with *Astragalus nakaianus* (GenBank accession no. NC028171.1) as the reference genome. The complete assembled genome was annotated via PGA (Qu et al. 2019).

The complete cp genome of *A. scaberrimus* (GenBank accession no. MW654102) has a circular form with a length of 123,492 bp, a 34% GC content and IR loss, which is common in Fabaceae, especially in Papilionoideae (Cai et al. 2008; Yi et al. 2020). A total of 113 genes were predicted for this cp genome, comprising 79 encoded proteins, 4 rRNAs and 30 tRNAs.

Phylogenetic analysis was performed on complete cp genomes of *A. scaberrimus* and other 29 related species in Fabaceae with two species in Polygalaceae as outgroups. The alignment was constructed by HomBlocks (Bi et al. 2018), and the evolutionary history was inferred using the maximum likelihood (ML) method by IQ-TREE 1.6.12 under the TVM + F + I + G4 model (Nguyen et al. 2015; Kalyaanamoorthy et al. 2017). Bootstrap (BS) values were calculated by UFBoot2 from 1000 replicates (Hoang et al. 2018), and the final output file was edited in MEGA X (Kumar et al. 2018). As expected, *A. scaberrimus* was grouped within *Astragalus* and formed a clade with *Astragalus laxmannii* with a 100% BS support value (Figure 1). The complete cp genome of *A. scaberrimus* can serve as a reference for future studies on molecular biology, evolution, population genetics, taxonomy and resource protection.

**Figure 1.**
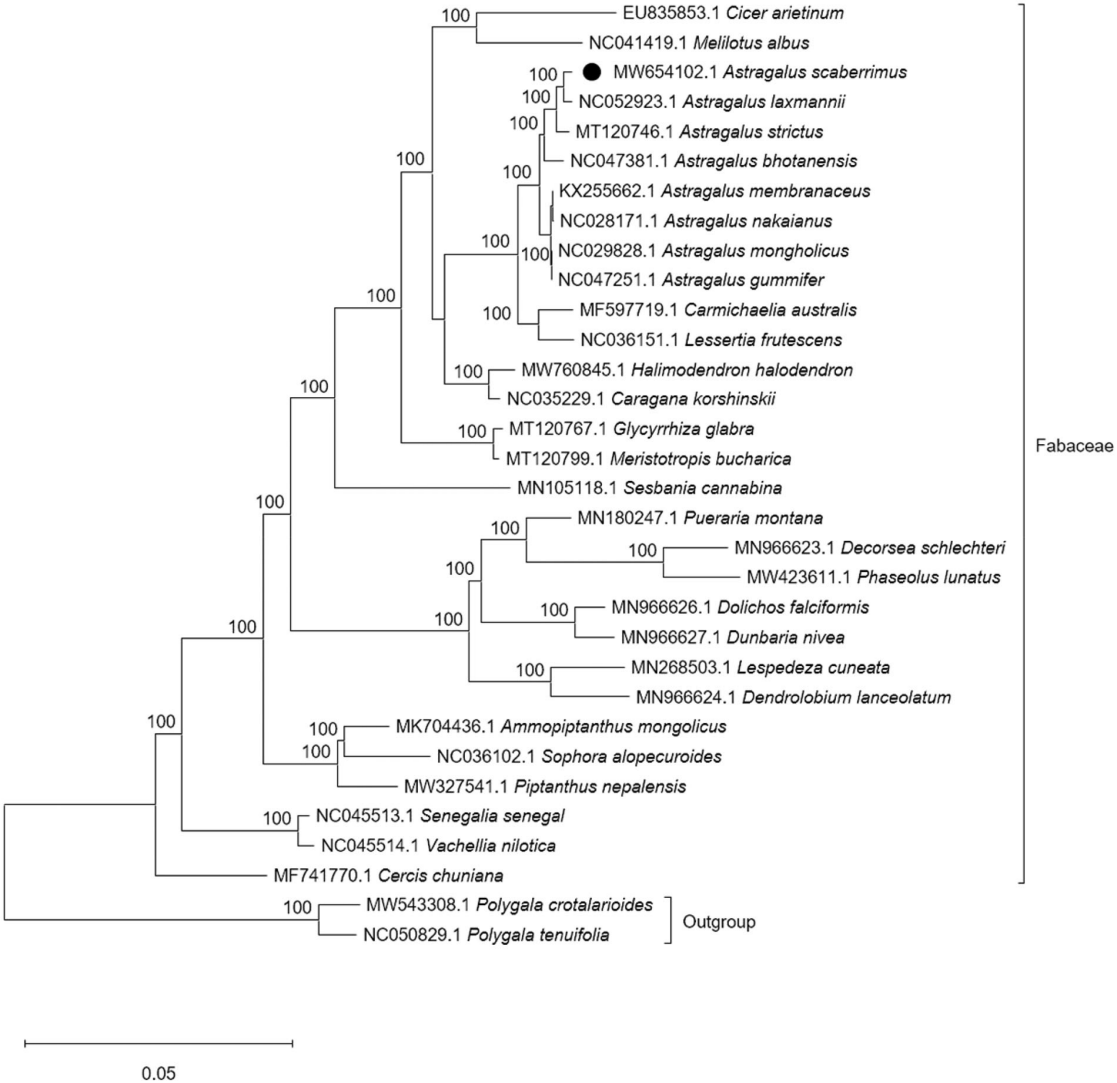
ML phylogenetic tree based on 32 species chloroplast genomes was constructed using IQ-TREE 1.6.12. Numbers on each node are bootstrap support values from 1000 replicates.

## Data Availability

The genome sequence data that support the findings of this study are openly available in GenBank of NCBI at (https://www.ncbi.nlm.nih.gov/nuccore/MW654102) under the accession no. MW654102. The associated BioProject, SRA, and Bio-Sample numbers are PRJNA725312, SRR14328337, and SAMN18875902, respectively.
